# 5-AzaC Facilitates Somatic Embryogenesis and Germination Across Two Embryogenic Lines in *Larix olgensis*

**DOI:** 10.3390/plants14182818

**Published:** 2025-09-09

**Authors:** Wenna Zhao, Yu Liu, Chen Wang, Yajing Ning, Chengpeng Cui, Hanguo Zhang, Meng Li, Shujuan Li

**Affiliations:** State Key Laboratory of Tree Genetics and Breeding, Northeast Forestry University, Harbin 150040, China

**Keywords:** *Larix olgensis*, 5-AzaC, somatic embryogenesis, methylation inhibitors

## Abstract

Long-term subculture of embryogenic callus leads to a decline in somatic embryogenesis and germination capacity, which may be associated with increased methylation levels. 5-Azacytidine (5-AzaC), a methylation inhibitor, modulates DNA methylation and is widely involved in regulating plant growth, development, and metabolism. In order to investigate the effect of 5-AzaC on somatic embryogenesis and germination in *Larix olgensis*, we supplemented the proliferation medium with different concentrations of 5-AzaC. The results showed that the addition of 5-AzaC inhibited the proliferation of embryogenic callus, with the proliferation of embryogenic line N2 completely inhibited at 100 μM, while that of embryogenic line N4 ceased at 20 μM. In contrast, treatment with 10 μM and 20 μM of 5-AzaC significantly increased the somatic embryo yield in both embryogenic lines, with the peak yield observed at 20 μM for embryogenic line N2 and at 10 μM for embryogenic line N4. Furthermore, the addition of 10 μM 5-AzaC effectively reduced the deformity rate during somatic embryo germination in embryogenic line N2 and N4, by 15.91% and 13.53%, respectively. These findings demonstrate that 5-AzaC can partially restore the somatic embryogenesis potential of embryogenic callus in *L. olgensis* under long-term subculture. Additionally, these results suggest that its effects may be both concentration-dependent and genotype-specific. The results provide a potential approach to mitigating the decline in embryogenic competence, while also demonstrating significant potential for large-scale propagation.

## 1. Introduction

*Larix olgensis* (*L. olgensis*) is primarily distributed in the alpine regions of northeastern and northern China [1]. As one of the three major coniferous timber species in northeast China, it is characterized by rapid growth and high resistance to environmental stress [2,3]. Somatic embryogenesis is considered the most efficient method for conifer propagation and represents an important method for the asexual reproduction of trees [4]. In recent years, research on somatic embryogenesis in *L. olgensis* has received considerable attention. A procedure for induction and somatic embryogenesis in *L. olgensis* has been established [5], providing a solid foundation for further studies and offering important theoretical and technical support for the conservation and utilization of this tree species. Somatic embryogenesis involves complex interactions among the plant growth regulators, explant source, medium composition, nitrogen and carbon sources, and in vitro culture conditions [6]. However, embryogenic callus in conifers gradually loses its embryogenic capacity during prolonged subculture and may transition into non-embryogenic callus [7,8,9]. Additionally, most of the somatic embryos that undergo maturation and germination develop into deformed buds.

DNA methylation levels are closely associated with the capacity for somatic embryogenesis and germination, playing a critical role in the regulation of gene expression during development [10]. Embryogenic callus exhibits lower DNA methylation levels compared to those in non-embryogenic callus, allowing more genes to remain transcriptionally active and thereby supporting successful somatic embryogenesis [11]. Studies have indicated that the decline in somatic embryogenesis potential during prolonged in vitro culture may be partly attributed to increased DNA methylation [12]. As a widely used DNA methylation inhibitor, 5-AzaC has facilitated extensive research into the role of DNA methylation in embryogenic development [13]. Different concentrations of 5-AzaC and treatment durations can alter the methylation patterns of genomic DNA, thereby influencing embryogenic maintenance and somatic embryogenesis [14,15,16,17]. For example, in a study focusing on *Cocos nucifera* L., treatment with 15 μM and 20 μM of 5-AzaC for three days significantly promoted early somatic embryo formation while maintaining low overall DNA methylation levels [18]. Similarly, 5-AzaC treatment promoted early somatic embryogenesis and reduced DNA methylation levels in *Dimocarpus longan* Lour. [19].

The restoration of somatic embryogenesis and germination potential is crucial in conifer breeding studies. Studies on the effects of 5-AzaC on somatic embryogenesis have been conducted in several conifer species, including *Picea omorika* (PANC) PURK. [20], hybrid larch (*Larix × eurolepis*) [21], *Pinus elliottii* × *Pinus caribaea* var. *hondurensis* [9], *Pinus pinaster* (Ait.) [8], *Taxodium* hybrid ‘Zhongshanshan’ [22], and *Cephalotaxus mannii* [23]. Results across these species indicate that the effects of 5-AzaC vary with concentration, either promoting or inhibiting the proliferation of embryogenic tissues and somatic embryogenesis. For instance, in a study of *Pinus pinaster* [8], 5 μM of 5-AzaC significantly reduced the fresh weight of the embryogenic callus, whereas 10 μM and 15 μM treatments improved somatic embryo maturation. In contrast, a study on the *Taxodium* hybrid ‘Zhongshanshan’ [22] reported that treatment with 5 μM of 5-AzaC enhanced the maturation rate of the callus and accelerated somatic embryo formation. Furthermore, a study based on two embryogenic cell lines (ELC02 and ELC05) of *Pinus elliottii* × *Pinus caribaea* var. *hondurensis* [9] demonstrated that treatment with 150 μM of 5-AzaC upregulated the expression of *SERK1* and *LEC1*, highlighting that the effect of 5-AzaC is also genotype-dependent. To date, the influence of 5-AzaC on somatic embryogenesis in *L. olgensis* has not been reported. Therefore, this study aims to investigate the effects of different concentrations of 5-AzaC on somatic embryogenesis in different embryogenic lines of *L. olgensis*. Our work aims to explore a method for mitigating the decline in somatic embryogenesis potential caused by long-term subculture, thereby supporting the large-scale propagation of conifers.

## 2. Results

### 2.1. The Selection of the Materials

#### 2.1.1. Microscopic Examination of Embryogenic Callus

**Figure 1:**
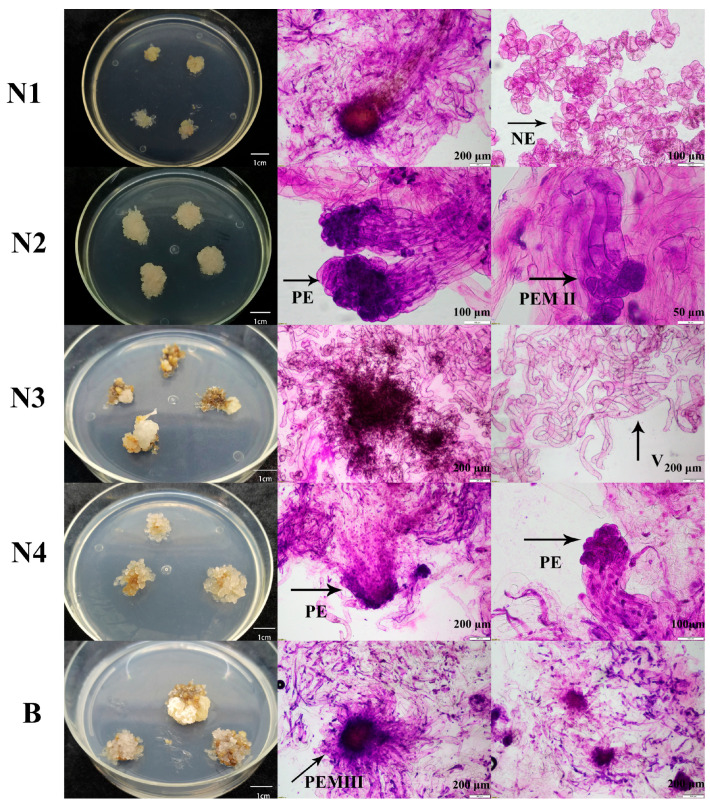
Microscopic examination of different embryogenic lines. Embryogenic cells N1–N4 were induced from genetic line 73-22 of *L. olgensis*, while embryogenic cells B were induced from genetic line 561 of *L. olgensis*. NE means non-embryogenic callus, PE means primary embryo, PEM II and PEM III mean proembryogenic mass II and III, and V means vacuolated, long-stalked cells.

Four embryogenic lines N1, N2, N3, and N4 and one embryogenic line B were induced from genetic lines 73-22 and 561 of *L. olgensis*, respectively (Figure 1). Embryogenic line N1 exhibited fine granulation, with many rounded embryogenic cell clusters lacking suspensory cells under microscopic observation. Following prolonged proliferative culture, the callus surface showed filamentous structures containing embryogenic suspensor cells. Embryogenic line N2 showed no browning during proliferation and displayed a denser texture. Microscopic examination revealed intact embryogenic structures, primarily at the proembryogenic mass (PEM) II (Roman numerals denote distinct developmental stages of the proembryogenic mass) and primary embryo (PE) stages. Embryogenic line N3 produced more translucent somatic embryos during proliferation. Subsequent microscopic examination revealed more vacuolated, long-stalked cells with a loss of embryogenic properties. After prolonged subculture, browning and death of the embryogenic callus occurred. Embryogenic line N4 exhibited central browning during the process of proliferation. Its surface proliferative tissue showed a reduced compactness, while microscopic examination confirmed that the embryogenic callus maintained its structural integrity, primarily at the PE stage. Embryogenic line B progressively lost its embryogenic properties during proliferation, accompanied by browning and eventual death. Microscopic observation showed that the embryogenic cells were predominantly at the PEM Ш stage. However, these cells exhibited a shrunken morphology. Based on these characteristics, embryogenic lines N1, N2, and N4 were selected for further somatic embryogenesis and regeneration potential screening.

#### 2.1.2. Screening of the Embryogenic Lines

**Figure 2:**
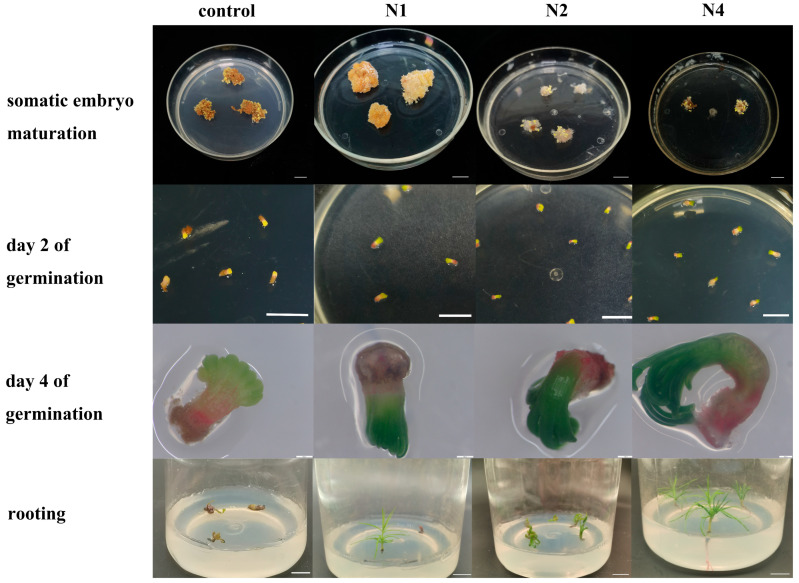
Somatic embryogenesis and germination of different embryogenic lines. The control was from embryogenic callus subcultured for 8 years. The scale bars for rows 1, 2, and 4 are 1 cm, while that for row 3 is 500 μm.

Somatic embryos can be observed following about 40 days of culture on maturation medium (Figure 2). The somatic embryos were separated and transferred onto germination medium. The germination morphology was assessed after two days. An embryogenic callus subcultured for 8 years was used as the control group. The control group exhibited a low number of somatic embryos, all of which exhibited morphological abnormalities. After two days of germination, these embryos showed no green pigmentation at the apex or red pigmentation at the base. After four days of germination, The meaning is as follow: the cotyledons showed partial yellowish-green discoloration, accompanied by browning at the base. The tissue displayed vitrification and eventually died. Embryogenic line N1 produced a limited number of somatic embryos. Apical–basal polarity was established after two days of germination. Cotyledon emergence occurred on day four of germination, followed by normal expansion and morphogenesis. Crucially, root meristem development was arrested after seven days of germination, preventing plantlet formation. Embryogenic lines N2 and N4 produced significantly more somatic embryos. Distinct apical–basal polarity was established after two days of germination in embryogenic lines N2 and N4, as observed in N1. Notably, only embryogenic line N4 achieved normal germination and formed intact plantlets. Based on their normal proliferation capacity and ability to produce mature somatic embryos, lines N2 and N4 were selected for subsequent studies.

### 2.2. The Effects of 5-AzaC on Proliferation and Somatic Embryogenesis

#### 2.2.1. The Effects of 5-AzaC on the Proliferation of Different Embryogenic Lines

**Figure 3:**
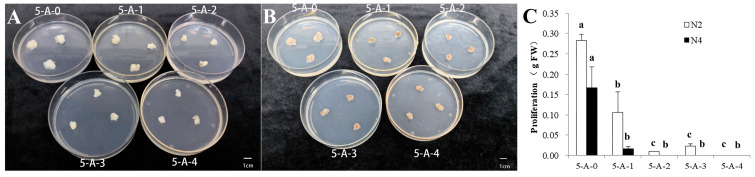
The effects of different concentrations of 5-AzaC on the proliferation of embryogenic callus. (**A**,**B**) Proliferation status of embryogenic lines N2 and N4 respectively; (**C**) histogram of proliferation. 5-A-0: 0 μM of 5-AzaC; 5-A-1: 10 μM of 5-AzaC; 5-A-2: 20 μM of 5-AzaC; 5-A-3: 50 μM of 5-AzaC; 5-A-4: 100 μM of 5-AzaC. The scale of the images is 1 cm. Different letters mean a significant difference in (**C**) (*p* < 0.05).

Following six months of proliferation, 0.1 g of fresh embryogenic callus from embryogenic lines N2 and N4 was transferred onto proliferative medium supplemented with different concentrations of 5-AzaC for 15 days. As shown in Figure 3A,B, the addition of 5-AzaC inhibited the proliferation of the embryogenic callus to varying degrees. The proliferation of the embryogenic callus was significantly reduced at all four 5-AzaC concentration compared to that for the control (treatment without 5-AzaC) (Figure 3C) (the results of the ANOVA and Duncan’s analysis are provided in Table S1). The proliferation of embryogenic line N2 completely stopped at 100 μM of 5-AzaC, whereas the proliferation of embryogenic line N4 completely stopped at a concentration of 20 μM of 5-AzaC.

#### 2.2.2. The Effects of 5-AzaC on Somatic Embryo Maturation

**Figure 4:**
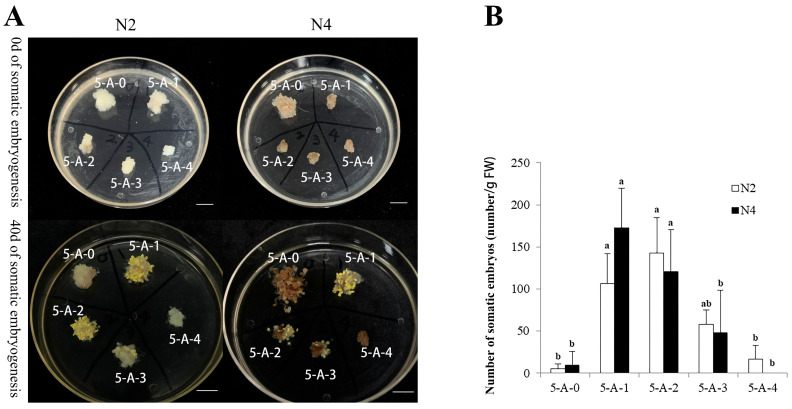
The effect of 5-AzaC on somatic embryogenesis. (**A**) The morphology of somatic embryogenesis under different 5-AzaC treatments; (**B**) the number of somatic embryos under different 5-AzaC treatments. 5-A-0: 0 μM of 5-AzaC; 5-A-1: 10 μM of 5-AzaC; 5-A-2: 20 μM of 5-AzaC; 5-A-3: 50 μM of 5-AzaC; 5-A-4: 100 μM of 5-AzaC. The scale of the images is 1 cm. Different letters mean a significant difference in (**B**) (*p* < 0.05).

As shown in Figure 4A (5-A-0), only several somatic embryos were observed following six months of the proliferation culture. This indicated that the potential for somatic embryogenesis in embryogenic lines N2 and N4 was significantly lower than that in the early proliferation phase (Figure 2). However, appropriate concentrations of 5-AzaC significantly increased the somatic embryo yield in both embryogenic lines (Figure 4B) (the results of the ANOVA and Duncan’s analysis are provided in Table S2). In embryogenic line N2, the number of somatic embryos initially increased but subsequently decreased with increasing treatment concentrations. The highest yield of somatic embryos occurred at the treatment concentration of 20 μM, which was 26.35-fold that in the control (treatment without 5-Azac). In embryogenic line N4, the somatic embryo yield reached a maximum at 10 μM of 5-AzaC (18.14-fold that in the control) and then decreased with an increasing concentration. No somatic embryos were observed at a concentration of 100 μM. In conclusion, 10 μM and 20 μM of 5-AzaC significantly increased the number of somatic embryos in both embryogenic lines.

#### 2.2.3. The Effect of 5-AzaC on Somatic Embryo Germination

**Figure 5:**
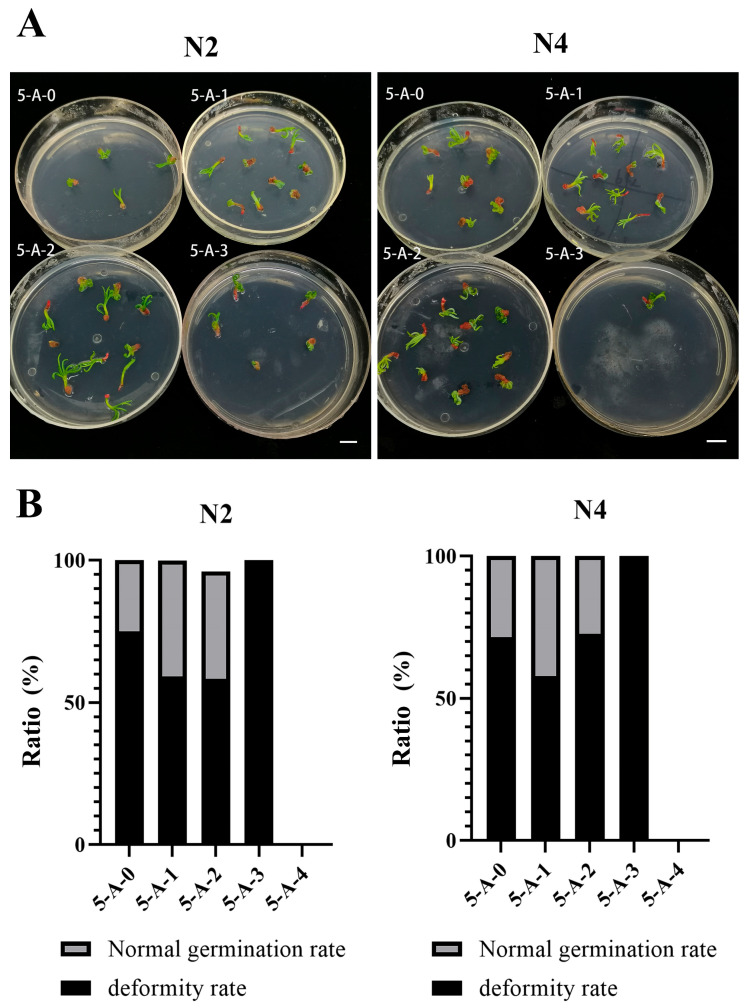
The effect of 5-AzaC on the germination of somatic embryos. (**A**) The morphology of the somatic embryos after germination under different 5-AzaC treatments; (**B**) rates of deformity and normality in the somatic embryos under different 5-AzaC treatments. 5-A-0-: 0 μM of 5-AzaC; 5-A-1: 10 μM of 5-AzaC; 5-A-2: 20 μM of 5-AzaC; 5-A-3: 50 μM of 5-AzaC; 5-A-4: 100 μM of 5-AzaC. The scale of the images is 1 cm.

Somatic embryos suitable for the germination experiment were produced only in the treatments with 5-AzaC concentrations of 0 μM, 10 μM, 20 μM, and 50 μM. As shown in Figure 5A, somatic embryos from these four treatments were capable of germination. Among them, a portion developed normal cotyledons and root systems, while others exhibited swelling and curling, followed by germination stagnation and failure to develop into intact plantlets. Appropriate concentrations of 5-AzaC reduced the germination deformity rate in the somatic embryos. As shown in Figure 5B, at concentrations of 10 μM and 20 μM, the deformity rate in embryogenic line N2 decreased by 15.91% and 16.67%, respectively. But the germination deformity rate of embryogenic line N4 was reduced by 13.53% only at a treatment concentration of 10 μM. Therefore, appropriate concentrations of 5-AzaC effectively reduced the deformity rate in the somatic embryos.

## 3. Discussion

### 3.1. The Decline in the Somatic Embryogenesis Potential Following Prolonged Subculture

In this study, embryogenic callus was induced from two genetic lines of *L. olgensis* (73-22 and 561), resulting in a total of five distinct embryogenic cell lines (N1, N2, N3, N4, and B) (Figure 1). Embryogenic lines N2 and N4 were mostly in the PEM Ш stage, whereas embryogenic lines N3 and B exhibited embryogenic loss, browning, and mortality during the subculture. This may have been related to the genotypes of the two genetic lines, as previously observed in *Larix principis-rupprechtii* [24]. Previous studies, including those by Jones and Van Staden [25] and Montalban et al. [26], substantiate that genotype is one of the main factors influencing somatic embryogenesis. We observed a significant reduction in the somatic embryogenesis potential in the two embryogenic lines N2 and N4 of *L. olgensis* after six months of the proliferation culture (Figure 4A). This finding is consistent with the loss of embryogenic competence reported in *Pinus pinaster* [8] and *Pinus koraiensis* [27], although embryogenic callus from these two conifer species ceased to produce somatic embryos or produced very few after 18 months and 12 months of culture, respectively. A more rapid loss (only 120 days) in the somatic embryogenesis potential was observed in *Brachypodium distachyon* [28]. In a study of sugarcane (*Saccharum* sp.), prolonged culture of the embryogenic tissues on medium containing 2,4-Dichlorophenoxyacetic acid (2,4-D) led to alterations in their starch, polyamine, and protein profiles, significantly reducing the efficiency of somatic embryogenesis [29]. Although 2,4-D was present in our proliferation medium, potentially contributing to the decline in the embryogenic potential in lines N2 and N4 in this study, we hypothesize, however, that the loss of embryogenic capacity cannot be attributed solely to 2,4-D. In our unpublished preliminary study, we substituted NAA for 2,4-D in the proliferation medium for the long-term subculture of embryogenic callus from *L. olgensis*. However, this substitution did not improve somatic embryogenesis compared to that in the cultures maintained on the 2,4-D-containing medium. Other factors, for example, hypoxic conditions, have been reported to impair metabolism and cause the loss of embryogenic potential in aged *Bactris gasipaes* cultures [30]. Studies in *Solanum betaceum* Cav. suggest that prolonged exposure to high sucrose concentrations may contribute to the loss of embryogenic capacity [31]. Additionally, the association between DNA methylation and somatic embryogenesis has been well documented [21]. All of these factors (plant growth regulator regimes, sugar signaling, hypoxia, and epigenetic modifications) may collectively or individually influence the loss of embryogenic competence in *L. olgensis*.

### 3.2. Restored Embryogenic Potential via 5-AzaC Treatment

During the induction of both somatic and zygotic embryogenesis in plants, DNA methylation is triggered. Crucially, DNA methylation is essential for somatic embryogenesis, mirroring its role in zygotic embryo development. Can methylation inhibitors restore the lost somatic embryogenesis capability in *L. olgensis* following long-term subculture? Our work found that 10 μM of 5-AzaC effectively increased the number of somatic embryos and reduced the deformation rate in the somatic embryos during the germination stage (Figure 4B and Figure 5B). Treatment with lower concentrations of 5-AzaC (5 μM) in the *Taxodium* hybrid ‘Zhongshanshan’ enhanced the somatic embryogenesis rate, the somatic embryo maturation rate, and the somatic embryo germination rate, consistent with the results of this study [22]. The positive effect of 5-AzaC on somatic embryogenesis may be due to the fact that this methylation inhibitor reduces the methylation levels of somatic-embryogenesis-related genes, thereby affecting the expression of these genes [32]. A transcriptome analysis of early somatic embryogenesis in *Dimocarpus longan* revealed that 5-AzaC takes part in metabolic regulation by changing the expression of genes related to somatic embryogenesis [19]. Examples include SERK kinases, which have important functions in somatic embryogenesis. In a study on *Ananas comosus*, 5-AzaC increased the expression of *AcSERK1* and promoted somatic embryogenesis [33]. However, the positive effect of 5-AzaC on somatic embryogenesis is not universal. In a study focusing on *Cucurbita pepo* L., the addition of 5-AzaC had no effect on somatic embryogenesis [16]. Moreover, the effect of 5-AzaC on the embryogenic callus is highly dependent on its concentration. Our work found that a concentration of 100 μM of 5-Azac completely inhibited the proliferation of embryogenic lines N2 and N4 (Figure 3C). This is consistent with the findings of Teyssier et al. [21] that adding 5-AzaC to the proliferation medium will inevitably affect the differentiation status of explants across plant species. The reason for the inhibition of proliferation may be that different concentrations of 5-AzaC may break DNA double strands and induce different degrees of cell death [34]. This study also revealed genotype-dependent responses to 5-AzaC treatment. The proliferation of embryogenic line N4 was completely inhibited at 20 μM of 5-AzaC, while that of embryogenic line N2 was inhibited at a higher concentration of 100 μM (Figure 3C). Moreover, the highest somatic embryo production occurred at 20 μM in embryogenic line N2 but at 10 μM in embryogenic line N4 (Figure 4B). Similar findings were reported in *Pinus elliottii* × *Pinus caribaea* var. *hondurensis*, where 150 μM of 5-AzaC upregulated the expression of *SERK1* and *LEC1* in the aged genotype ECL05, thereby positively influencing its embryogenic morphology. In contrast, 5-AzaC exhibited the opposite response in the genotype ECL02 [9]. These results further support the concept that the effect of 5-AzaC on somatic embryogenesis in *L. olgensis* is genotype-dependent. Although both our results and the published findings suggest that the effects of 5-AzaC may be genotype-dependent, this conclusion remains to be validated across a broader range of embryonic lines, as both studies were initiated using only two embryogenic lines. Nonetheless, our findings provide a potential approach to mitigating the decline in embryogenic competence caused by prolonged subculture while also demonstrating significant potential for its large-scale propagation.

## 4. Materials and Methods

### 4.1. The Experimental Materials

**Figure 6:**
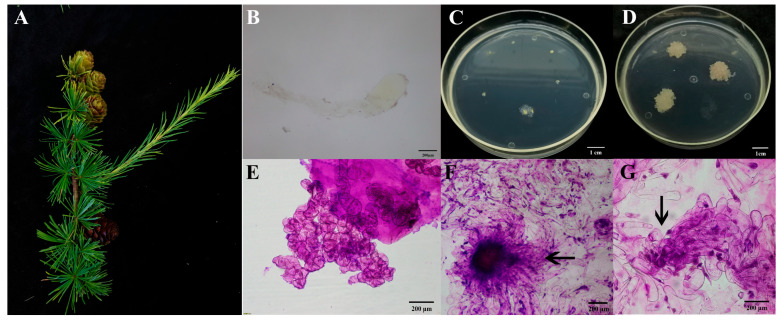
The process of embryogenic callus induction. (**A**) The explants collected in July 2023; (**B**) an immature zygotic embryo; (**C**) the early induction stage of the embryonic callus; (**D**) the proliferation of the embryogenic callus; (**E**) embryogenic cells without suspensory cells; (**F**,**G**) the black arrow indicates the embryogenic callus in the PEM III stage.

On July 1 2023, large sporophyte cones of genetic lines 73-22 and 561 of *L. olgensis* were collected from the Mengjiagang Experimental Forestry in Jiamusi City, Heilongjiang Province, China (Figure 6A). The cones were harvested, sealed into plastic bags, and kept in an insulated container with ice during transport to the laboratory, where they were subsequently stored at 4 °C. Intact immature seeds were chosen as the explants (Figure 6B). Sterilization was performed by referring to the methods of Song et al. [5]. The immature embryos were cultured on induction medium (BM medium supplemented with 1.0 g·L^−1^ of glutamine, 0.5 g·L^−1^ of acid-hydrolyzed casein, 1 g·L^−1^ of inositol, 5.4 g·L^−1^ of agar, 25 g·L^−1^ of sucrose, 1.0 mg·L^−1^ of 2,4-D, 0.5 mg·L^−1^ of 6-benzylaminopurine (6-BA), and 0.3 mg·L^−1^ of kinetin (KT), at a pH of 6.0 ± 0.02). The materials were maintained at 25 ± 1 °C in the dark. After 20 days of induction, colorless and transparent tissue grew from the endosperm cuts of the explants in the medium (Figure 6C,D). Embryogenic cells without suspensor cells and embryogenic cells at the PEM Ш stage were observed after Carbol fuchsin staining under a microscope (Figure 6E–G). The embryogenic callus induced was cultured on the proliferation medium (BM medium supplemented with 1.0 g·L^−1^ of glutamine, 0.5 g·L^−1^ of acid-hydrolyzed casein, 1 g·L^−1^ of inositol, 5.4 g·L^−1^ of agar, 25 g·L^−1^ of sucrose, 0.1 mg·L^−1^ of 2,4-D, 0.05 mg·L^−1^ of 6-BA, and 0.03 mg·L^−1^ of KT, with a pH of 6.0 ± 0.02) for subsequent study.

### 4.2. Experimental Methods

#### 4.2.1. The Selection of the Experimental Materials

Well-developed embryogenic callus was selected, stained with Carbol fuchsin reagent, and then examined under a microscope to observe its morphology. Embryogenic callus (0.15 g per block) from the different embryogenic lines was cultured on the proliferation medium. After 15 days of stable growth in this medium, the embryogenic callus of the different embryogenic lines of *L. olgensis* was transferred onto transition medium (1/4 BM medium supplemented with 0.5 g·L^−1^ of glutamine, 0.25 g·L^−1^ of acid-hydrolyzed casein, 10 g·L^−1^ of inositol, 5.4 g·L^−1^ of agar, and 60 g·L^−1^ of sucrose, at a pH of 6.0 ± 0.02) and cultured for 10 days, after which point it was transferred onto somatic embryo maturation medium (BM medium supplemented with 0.5 g·L^−1^ of glutamine, 0.25 g·L^−1^ of acid-hydrolyzed casein, 10 g·L^−1^ of inositol, 2 g·L^−1^ of agar substitute gelling agent, 25 g·L^−1^ of sucrose, 80 g·L^−1^ of Polyethylene glycol 6000, and 20 mg·L^−1^ of abscisic acid, with a pH of 6.0 ± 0.02) and cultured in the dark at 25 ± 1 °C. After 40 days of culture on the somatic embryo maturation medium, the somatic embryos were transferred onto germination medium (1/2 MS medium, supplemented with 6.5 g·L^−1^ of plant agar and 30 g·L^−1^ of sucrose, with a pH of 6.0 ± 0.02). The morphology of the somatic embryos as well as their germination status at 2 days and 4 days and their subsequent rooting status were compared with these properties in embryogenic callus subcultured for 8 years. Based on this comparison, embryogenic material suitable for subsequent study was selected.

#### 4.2.2. The Effect of 5-AzaC on Somatic Embryogenesis

5-AzaC concentrations of 0 μM (5-A-0), 10 μM (5-A-1), 20 μM (5-A-2), 50 μM (5-A-3), and 100 μM (5-A-4) were added to the proliferation medium. For each treatment, about 0.1 g of the embryogenic callus from every embryogenic line screened in Section 4.2.1 was selected. After 14 days of treatment, the callus was weighed to calculate the proliferation. It was then transferred entirely onto maturation medium, and the number of somatic embryos was counted after 40 days of culture. Finally, all somatic embryos were transferred onto germination medium, and the germination rate and the deformity rate were assessed after 10 days. This experiment was repeated three times to analyze the effects of different 5-AzaC concentrations on the proliferation, somatic embryo yield, and somatic embryo germination in the different embryogenic lines.

### 4.3. The Statistical Analysis

The following parameters were calculated to evaluate the treatment effects. Proliferation (g) = *FW*_after_ − *FW*_before_; *FW*_before_ means the fresh weight of the embryogenic callus before inoculation, and *FW*_after_ means the fresh weight of the embryogenic callus after proliferation. Somatic embryo yield (number∙g^−1^) = *N*_SE_/*FW*_maturation_; *N*_SE_ means the total number of somatic embryos counted, and *FW*_maturation_ means the fresh weight of the callus transferred onto the maturation medium. Germination rate (%) = *N*_germination_/*N*_inoculated_ × 100; *N*_germination_ means the number of germinated somatic embryos, and *N*_inoculated_ means the total number of somatic embryos inoculated onto the germination medium. Deformity rate (%) = *N*_deformed_/*N*_inoculated_ × 100; *N*_deformed_ means the number of deformed germinated somatic embryos, and *N*_inoculated_ means the number of germinated somatic embryos. The statistical analysis of the proliferation and number of somatic embryos was based on three biological replicates. However, some treatments produced very few somatic embryos. Replication was therefore not possible for those cases. The germination rate and the deformity rate were calculated instead using all of the somatic embryos obtained from the three replicates. The experimental data are presented as the mean ± standard error (SE) and were analyzed in SPSS 25.0 using an ANOVA, followed by Duncan’s multiple range test. The significance level was set at *p* < 0.05.

## 5. Conclusions

In this study, two embryogenic lines of *L. olgensis* (N2 and N4) suitable for subculture and somatic embryogenesis were successfully induced and selected. However, both lines exhibited a decline in embryogenic competence after six months of subculture. Treatment with varying concentrations of 5-AzaC revealed that all tested concentrations inhibited the proliferation of the embryogenic callus. Notably, complete inhibition of proliferation was observed at 20 μM for embryogenic line N4, in contrast to embryogenic line N2, which required a much higher concentration of 100 μM for the same effect. However, 10 μM and 20 μM of 5-AzaC significantly increased the somatic embryo yield in both the N2 and N4 lines, with the maximum production occurring at 10 μM for embryogenic line N4 and at 20 μM for embryogenic line N2. Furthermore, 10 μM of 5-AzaC consistently reduced the somatic embryo germination deformity rates in both embryogenic lines. A key finding of this study is the genotype-dependent response to 5-AzaC treatment, as evidenced by the differential optimal concentrations for somatic embryogenesis between lines N2 and N4. But this finding requires validation across a broader range of embryogenic lines. However, the dynamics of the methylation levels during the somatic embryogenesis process remain uncharacterized. In order to elucidate the mechanisms of 5-AzaC facilitated somatic embryogenesis in *L. olgensis*, future work should combine (1) a methylation analysis of embryogenic calli at different subculture durations, (2) methylation profiling across distinct somatic embryo developmental stages, and (3) an assessment of the methylation patterns in embryogenic calli treated with varying 5-AzaC concentrations, aiming to elucidate the mechanisms underlying the partial restoration of embryogenic competence.

## Data Availability

All data are available in the manuscript and the Supplemental Materials.

## Supplementary Materials

The following supporting information can be downloaded at: https://www.mdpi.com/article/10.3390/plants14182818/s1, Table S1: ANOVA and Duncan’s test of proliferation across different treatments; Table S2: ANOVA and Duncan’s test of somatic embryo yield across different treatments.
